# Enhanced Virus Translation Enables miR-122-Independent Hepatitis C Virus Propagation

**DOI:** 10.1128/jvi.00858-21

**Published:** 2023-06-20

**Authors:** Mamata Panigrahi, Michael A. Palmer, Joyce A. Wilson

**Affiliations:** a Department of Biochemistry, Microbiology and Immunology, College of Medicine, University of Saskatchewan, Saskatoon, Saskatchewan, Canada; University of Southern California

**Keywords:** HCV, miR-122-independent replication, viral translation, 5′ UTR, genome stability, 5′ untranslated region, hepatitis C virus, miR-122, translation

## Abstract

The 5′ untranslated region (UTR) of the hepatitis C virus (HCV) genome forms RNA structures that regulate virus replication and translation. The region contains an internal ribosomal entry site (IRES) and a 5′-terminal region. Binding of the liver-specific microRNA (miRNA) miR-122 to two binding sites in the 5′-terminal region regulates viral replication, translation, and genome stability and is essential for efficient virus replication, but its precise mechanism of action is still unresolved. A current hypothesis is that miR-122 binding stimulates viral translation by facilitating the viral 5′ UTR to form the translationally active HCV IRES RNA structure. While miR-122 is essential for detectable replication of wild-type HCV genomes in cell culture, several viral variants with 5′ UTR mutations exhibit low-level replication in the absence of miR-122. We show that HCV mutants capable of replicating independently of miR-122 display an enhanced translation phenotype that correlates with their ability to replicate independently of miR-122. Further, we provide evidence that translation regulation is the major role for miR-122 and show that miR-122-independent HCV replication can be rescued to miR-122-dependent levels by the combined impacts of 5′ UTR mutations that stimulate translation and by stabilizing the viral genome by knockdown of host exonucleases and phosphatases that degrade the genome. Finally, we show that HCV mutants capable of replicating independently of miR-122 also replicate independently of other microRNAs generated by the canonical miRNA synthesis pathway. Thus, we provide a model suggesting that translation stimulation and genome stabilization are the primary roles for miR-122 in promoting HCV.

**IMPORTANCE** The unusual and essential role of miR-122 in promoting HCV propagation is incompletely understood. To better understand its role, we have analyzed HCV mutants capable of replicating independently of miR-122. Our data show that the ability of viruses to replicate independently of miR-122 correlates with enhanced virus translation but that genome stabilization is required to restore efficient HCV replication. This suggests that viruses must gain both abilities to escape the need for miR-122 and impacts the possibility that HCV can evolve to replicate outside the liver.

## INTRODUCTION

Hepatitis C virus (HCV) infects almost 71 million people worldwide (1), and chronic HCV can lead to liver cirrhosis and hepatocellular carcinoma (2, 3). HCV has a positive-strand RNA genome of 9.6 kb in length that contains a polyprotein coding region flanked by structured 5′ and 3′ untranslated regions (UTRs) that regulate virus translation and replication. Nucleotides 40 to 372 of the viral 5′ UTR, including stem-loop 2 (SLII) to SLIV, comprise the HCV internal ribosomal entry site (IRES) that directs cap-independent virus translation initiation (4–6). The 5′-terminal 42 nucleotides comprise SLI and two binding sites, site 1 (S1) and S2, for a host liver-specific microRNA (miRNA), miR-122, whose annealing is required for efficient viral propagation (7–11). The complement of the 5′-terminal region, the 3′ UTR of the negative strand, also contains RNA stem-loops that are important for viral replication (12, 13). Thus, the extreme 5′ end of the viral genome is a multifunctional regulatory sequence that harbors structures required for both viral replication and translation.

MicroRNAs are short, noncoding RNAs (20 to 24 nucleotides in length) that normally downregulate cellular gene expression and facilitate RNA decay (14–16). During miRNA suppression, endogenously expressed miRNAs, in association with one of the host Argonaute proteins (Ago 1, 2, 3, and 4), form the core element of the RNA-induced silencing complex (RISC) and target mRNAs for translation silencing and degradation through sequence complementarity within the 3′ UTR (17). However, in an unusual role for a miRNA, annealing of miR-122 to the 5′ UTR of the HCV genome promotes viral propagation and RNA accumulation (9, 10). Proposed functions for miR-122 include stimulation of virus translation (18–20), protecting the viral genome from cellular exonucleases (XRN1 and XRN2) and pyrophosphatases DOM3Z and DUSP11 (21–25), and directly promoting viral genomic RNA replication (26). However, the relative impact of each function on overall viral propagation remains unknown (27).

**FIG 1:**
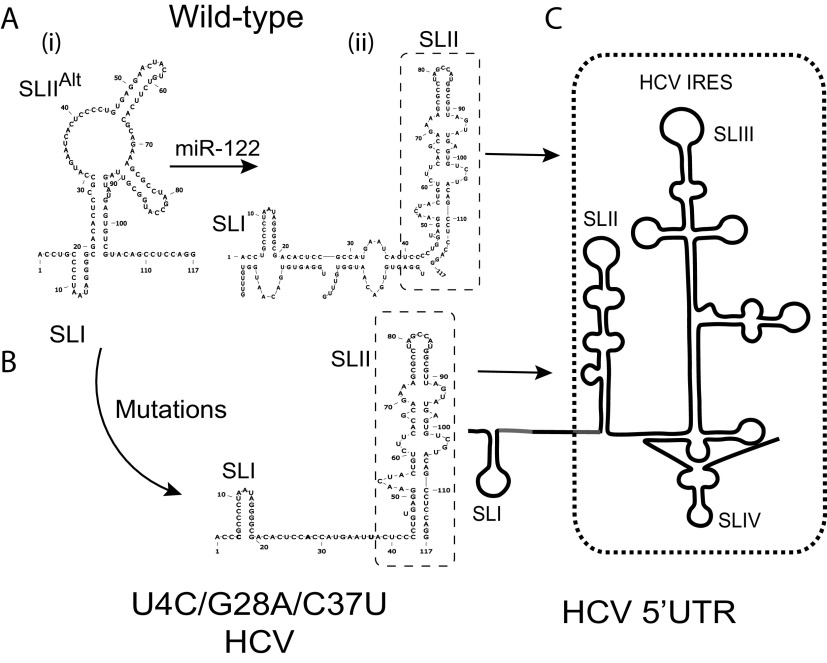
Hypothetical model for miR-122 and 5′ UTR mutation-induced RNA structure modifications leading to IRES activation. (A) Structure prediction of J6/JFH-1 p7Rluc2a HCV (i) without and (ii) with miR-122. Without miR-122, the first 117 nucleotides of HCV 5′ UTR are predicted to form a noncanonical structure with SLI and an altered SLII (SLII^Alt^), whereas miR-122 annealing to the viral 5′ UTR is predicted to form the canonical SLII structure. (B) The 5′ UTR of a mutant HCV (U4C/G28A/C37U) capable of miR-122-independent replication is also predicted to form the canonical SLII structure in the absence of miR-122. (C) Structure of the active HCV IRES including SLII.

An emerging hypothesis suggests a role for miR-122 as an RNA chaperone that modulates the structure of the 5′ UTR and the activity of the HCV IRES (10, 11, 28, 29). RNA structure predictions suggest that nucleotides 1 to 42 preceding viral IRES may modify the thermodynamics of the RNA IRES structure and that annealing of miR-122 may shift the thermodynamics to favor the formation of the active IRES RNA structure (Fig. 1). While this hypothesis remains to be confirmed using biophysical or biochemical methods, indirect support stems from the analysis of HCV mutants capable of miR-122-independent replication (10, 11). The first model of miR-122-independent replication was a bicistronic subgenomic RNA that contains an encephalomyocarditis virus (EMCV) IRES and suggested that altered translation regulation alleviates the need for miR-122 for viral propagation (30). Moreover, full-length HCV genomes having nucleotide substitutions or deletions in the 5′-terminal region have been found that exhibit miR-122-independent replication. While replication is detectable, all of the models of miR-122-independent replication support relatively inefficient replication and are still dependent on miR-122 for wild-type (WT) levels of replication (10, 31–35). The mechanisms of miR-122-independent replication of these HCV variants are still unknown. Prediction software suggests that the 5′ UTR RNA of the mutants has a greater propensity to form the active HCV IRES even in the absence of miR-122 (10, 11) (Fig. 1), but a report has also highlighted the possibility of binding of other microRNAs to the 5′ UTR of the viral genome (36).

In this study, we have developed new mutant HCV variants capable of replicating in the absence of miR-122 and used these and previously published mutants to investigate the mechanism of miR-122-independent replication. Our results suggest that mutants capable of miR-122-independent replication display enhanced translation activity compared to the wild-type HCV in the absence of miR-122 and that the translation efficiency is linked with miR-122-independent replication efficiency. We also characterized the relative contributions of translation and genome stability on miR-122-independent HCV replication and found that translation stimulation is essential for HCV propagation and that enhanced translation and genome stabilization can rescue wild-type levels of HCV replication in the absence of miR-122. Finally, we confirmed that HCV mutants capable of replicating independently of miR-122 also replicate independently of other miRNAs generated by Drosha and the canonical miRNA biogenesis pathways. Thus, our data support the notion that miR-122 has roles in both viral translation regulation and genome stabilization and suggest that these two mechanisms of action are sufficient to support wild-type levels of replication.

## RESULTS

### Some HCV genomes having point mutations across the miR-122 binding sites can replicate independently of miR-122.

**FIG 2:**
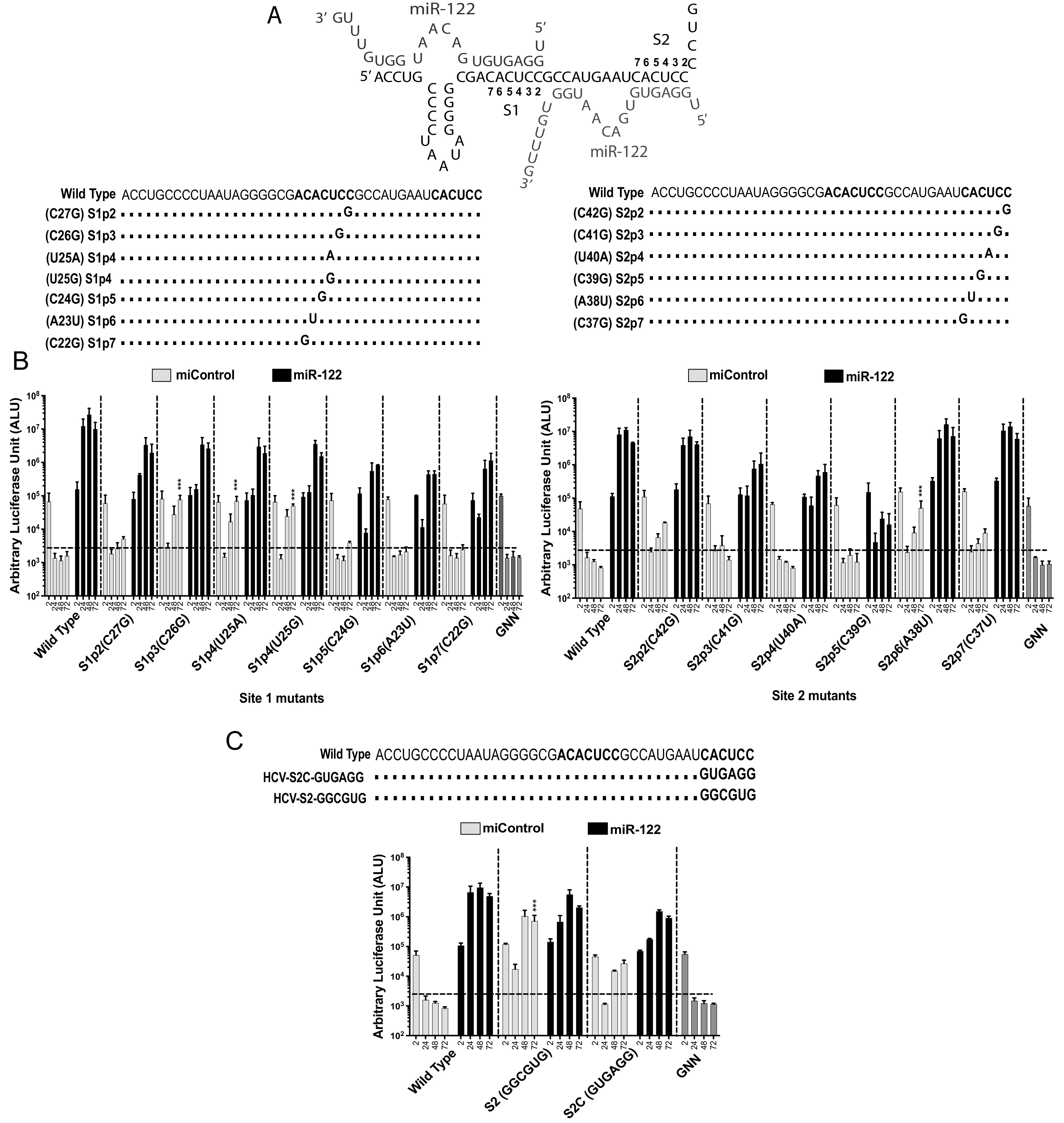
Some mutations to nucleotides within miR-122 binding sites 1 and 2 allow viral propagation in the absence of miR-122. (A) HCV 5′ UTR and the location and name of mutant viruses with sequence changes within miR-122 binding sites 1 and 2. “S” stands for miR-122 binding site (1 or 2) and “p” stands for the position in the binding site (1 to 7). (B) Replication of site 1 and site 2 mutant HCV J6/JFH-1 (p7Rluc2a) RNA in Huh 7.5 miR-122 knockout cells with a control miRNA (miControl) or with miR-122. *Renilla* luciferase was assessed at 2 h, 24 h, 48 h, and 72 h postelectroporation as an indicator of viral propagation. The dark gray and the light gray bars represent miR-122-dependent and -independent replication, respectively. (C) Replication of J6/JFH-1 (p7Rluc2a) HCV-S2-GGCGUG, and HCV-S2C-GUGAGG mutant RNA in Huh 7.5 miR-122 knockout cells with a control miRNA (miControl) or with miR-122. *Renilla* luciferase was assessed at 2 h, 24 h, 48 h, and 72 h postelectroporation as an indicator of viral propagation. The dark gray and the light gray bars represent miR-122-dependent and -independent replication, respectively. miR-122-independent replication of HCV mutant RNA was compared to that of the wild-type HCV RNA, with miControl as a control. All data are presented as the averages of three or more independent experiments. Error bars indicate the standard deviations of the means, and asterisks indicate significant differences. The significance was determined by using one-way analysis of variance (ANOVA) (***, *P* < 0.001).

Several studies have used viruses having mutations to the miR-122 binding sites to investigate the impact of miR-122 on virus replication (24, 36, 37). For example, we and others have used S1 position 3 (S1p3) and S1p4 (C26G and U25A) mutants to study the impact of abolishing miR-122 annealing. In addition, other studies have used an array of miR-122 binding site mutants (S2p1, -p2, -p3, -p4, and -p5) to determine the impact of miR-122 annealing at each nucleotide (37) and on the ability of viruses to replicate and sometimes revert (38). However, these mutants have not been assessed in transient replication assays for their impact on miR-122-dependent and miR-122-independent replication. In light of more recent evidence that point mutations at miR-122 binding sites facilitate miR-122-independent replication of HCV, we wanted to revisit these mutants to assess whether they are capable of miR-122-independent replication (10, 31). To investigate whether mutations in the miR-122 binding sites alter virus translation or allow the virus to replicate in the absence of miR-122, we mutated each nucleotide of both the miR-122 binding sites individually to their complementary nucleotide and tested for virus replication in the absence of miR-122 in Huh 7.5 miR-122 knockout (KO) cells. Altogether, we created 12 mutants: 6 for site 1, S1p2 to S1p7, and 6 for site 2, and S2p2 to S2p7 (Fig. 2A). Replication of these mutants was also tested in the presence of miR-122 to verify their replication efficiency when one miR-122 site was bound. We first noticed that C26G, also known as S1p3 (24), allows for miR-122-independent replication. This mutation has been used frequently to model abolition of miR-122 binding to S1, but our data indicate an additional impact on the miR-122-independent replication ability of this genome and suggest that additional interpretation might be necessary for experiments using this mutant virus. In addition, U25A (S1p4) and A38U (S2p6) also induced miR-122-independent replication. That U25A stimulated miR-122-independent replication was interesting since U25C had previously been shown to also have this effect (31). This prompted us to also test U25G, and we observed that it also induced miR-122-indepenedent replication. Thus, any nucleotide at position 25 except the wild-type U allows for miR-122-independent replication.

### miR-122 site 2 mutations allow HCV to replicate independently of miR-122.

Mutation of miR-122 binding site 2 from CACUCC to GGCGUG (HCV-S2-GGCGUG) was also reported to enable efficient miR-122-independent replication (34). In this mutant, all positions in S2 except for p5 were mutated, suggested that site 2 binding of miR-122 is dispensable for HCV propagation and that mutation can rescue miR-122-independent replication. In support of our model that miR-122-independent replication is facilitated by enhanced translation, we found HCV-S2-GGCGUG to exhibit enhanced translation ability compared with that of the virus having a wild-type 5′ UTR (Fig. 2C). To test the role of abolishing miR-122 binding site 2, we mutated each nucleotide of miR-122 binding site 2 to its complementary nucleotide (CACUCC to GUGAGG) (HCV-S2C) and tested its replication and translation in Huh 7.5 miR-122 binding KO cells. HCV-S2C also replicated independently of miR-122 (Fig. 2C) but about 10-fold less efficiently than HCV-S2 (Fig. 2C). Thus, mutations within the S2 region modulate the ability of the 5′ UTR to regulate virus dependence on miR-122.

### Impact of 5′ UTR point mutations on predicted RNA structures.

**FIG 3:**
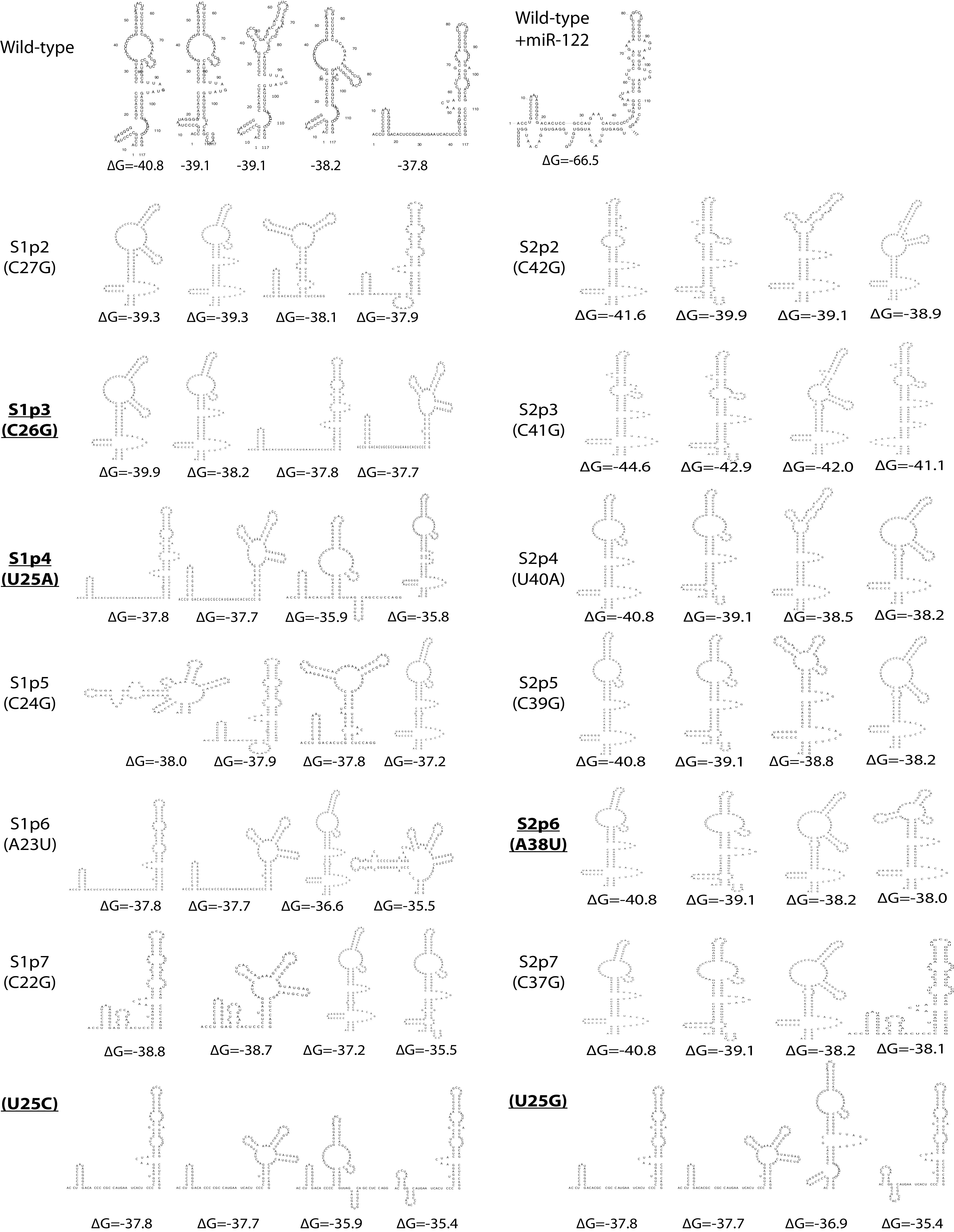
Structure prediction models of HCV J6/JFH-1 (p7Rluc2a) wild type and miR-122 binding site 1 and site 2 mutants. The sequences from nucleotides 1 to 117 of wild-type HCV and these mutants were analyzed using the “RNA structure” online tool, and the 4 lowest free-energy structure models are presented. These structures are prediction models and have not been experimentally validated.

We and others have also found that miR-122 annealing to the HCV 5′ terminus stabilizes the viral genomic RNA and stimulates virus translation, and we determined that translation stimulation as the primary mechanism by which miR-122 promotes HCV propagation (11, 29). In addition, previous *in silico* RNA structure predictions and biochemical analyses suggest that the 5′ UTR RNA forms a dynamic structure and that miR-122 shifts the folding equilibrium to favor the formation of the active IRES (Fig. 1) (10, 11, 28). Finally, we found that mutants exhibiting miR-122-independent replication are predicted to form the active IRES structure even in the absence of miR-122 (10) and that altered translation regulation enables miR-122-independent replication of bicistronic HCV constructs (30). Based on this, we hypothesized that viruses with 5′ UTR mutations that enable miR-122-independent replication would have enhanced translation and be predicted to have active IRES structures, and mutants incapable of miR-122-independent replication would translate less efficiently and be predicted to have less propensity to form the active IRES structure. In support of this hypothesis, we used RNA structure-predictive software (39) to analyze the possible structures of the mutant 5′ UTR sequences. The predicted 5′ UTR RNA structures of mutant viruses that could replicate independently of miR-122 (Fig. 3), the U25A, U25G, and U25C mutants, found to replicate independently of miR-122 in several previous studies (10, 31), favor formation of the active IRES structure, and the C26G mutant can form the active IRES. In contrast, the A38U mutant replicated independently of miR-122 but was not predicted to form the active IRES, suggesting the mutations at site 2 might function differently. However, these structures are predictions only and thus should be interpreted with caution.

### 5′ UTR mutations that induce miR-122-independent replication also stimulate viral translation.

**FIG 4:**
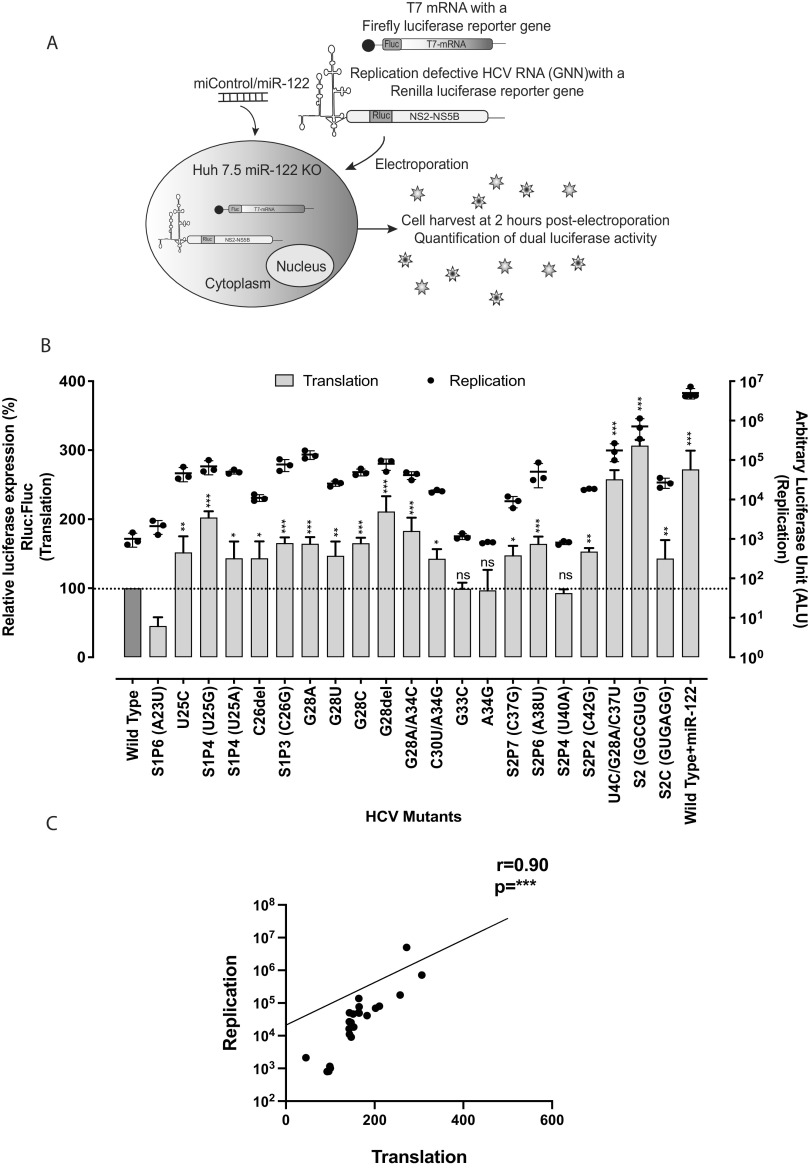
HCV mutants replicating independently of miR-122 have higher translation efficiency than wild-type HCV, and miR-122-independent translation potency correlates with miR-122-independent replication efficiency. (A) Illustration of the translation assay method. Replication-defective *Renilla* reporter J6/JFH-1 (p7Rluc2a) HCV RNA and a firefly reporter mRNA were coelectroporated into Huh 7.5 miR-122 knockout cells. Cells were harvested 2 h postelectroporation, and dual-luciferase activity was measured. (B) Comparison of translation and replication efficiencies of HCV mutants and wild-type virus in Huh 7.5 miR-122 knockout cells. The left *y* axis and bars represent HCV RNA translation, whereas the right *y* axis and the dots represent HCV RNA replication. For replication of mutant and wild-type HCV, viral RNA was electroporated into Huh 7.5 miR-122 knockout cells and cells were harvested 72 h postelectroporation. *Renilla* luciferase was measured to analyze viral propagation. (C) Correlation of HCV RNA translation and replication was analyzed using nonparametric Spearman correlation. All data are presented as the averages of three or more independent experiments. Error bars indicate the standard deviations of the means, and asterisks indicate significant differences. The significance was determined by using one-way ANOVA for translation efficiency and t-distribution for correlation (*, *P* < 0.033; **, *P* < 0.002; ***, *P* < 0.001).

To test our hypothesis that 5′ UTR mutations that enable miR-122-independent replication would enhance translation efficiency compared to that of the wild-type virus, we compared the translation efficiency of mutant genomes capable or incapable of miR-122-independent replication with that of wild-type viral genomes in Huh 7.5 miR-122 KO cells. We assessed mutant genomes from this (Fig. 2A) and other (10, 31, 34) studies. To focus on virus translation in this assay, we assessed *Renilla* luciferase (RLuc) expression from replication-defective reporter virus mutants (Rluc GNN) and a firefly luciferase (Fluc) mRNA (T7 mRNA) as an internal control. The *Renilla*-to-firefly luciferase ratio was measured to calculate the translation efficiency and is expressed as percent luciferase expression relative to the translation by wild-type viral RNA (Fig. 4A).

In support of our hypothesis, the translation assays showed that HCV mutants capable of replicating independently of miR-122 displayed higher translation efficiency than wild-type virus and mutants incapable of miR-122-independent replication (Fig. 4B). The level of enhanced translation also correlated with the efficiency of miR-122-independent replication in Huh 7.5 miR-122 KO cells (*r* = 0.9; *P* < 0.001) (Fig. 4C). For example, the U4C/G28U/C37U and HCV-S2-GGCGUG mutants exhibited the highest translation efficiency among the mutants and the highest miR-122-independent replication efficiency, mutants with intermediate translation efficiency (G28A, G28del, and G28C) and the U25C mutant exhibited intermediate miR-122-independent replication, and mutants that exhibited lower translation than other mutants (C26del, G28U, and C30U/A34G) replicated poorly in the absence of miR-122. Finally, the translation efficiencies of mutants incapable of replicating independently of miR-122 (A34G and G33C) were similar to that of wild-type virus. The result supports our hypothesis that enhanced translation efficiency by 5′ UTR mutant viruses allows HCV to replicate independently of miR-122 and suggest a role for miR-122 in viral translation regulation. In addition, mutation of miR-122 binding site 2 from CACUCC to GGCGUG (HCV-S2-GGCGUG) (34) and CACUCC to GUGAGG (HCV-S2-GGCGUG) also enhanced translation ability compared with that of the virus having a wild-type 5′ UTR (Fig. 4B), suggesting that the S2 region modulates the ability of the 5′ UTR to regulate virus translation and virus dependence on miR-122.

### Mutations do not enhance virus translation and replication when miR-122 is bound.

**FIG 5:**
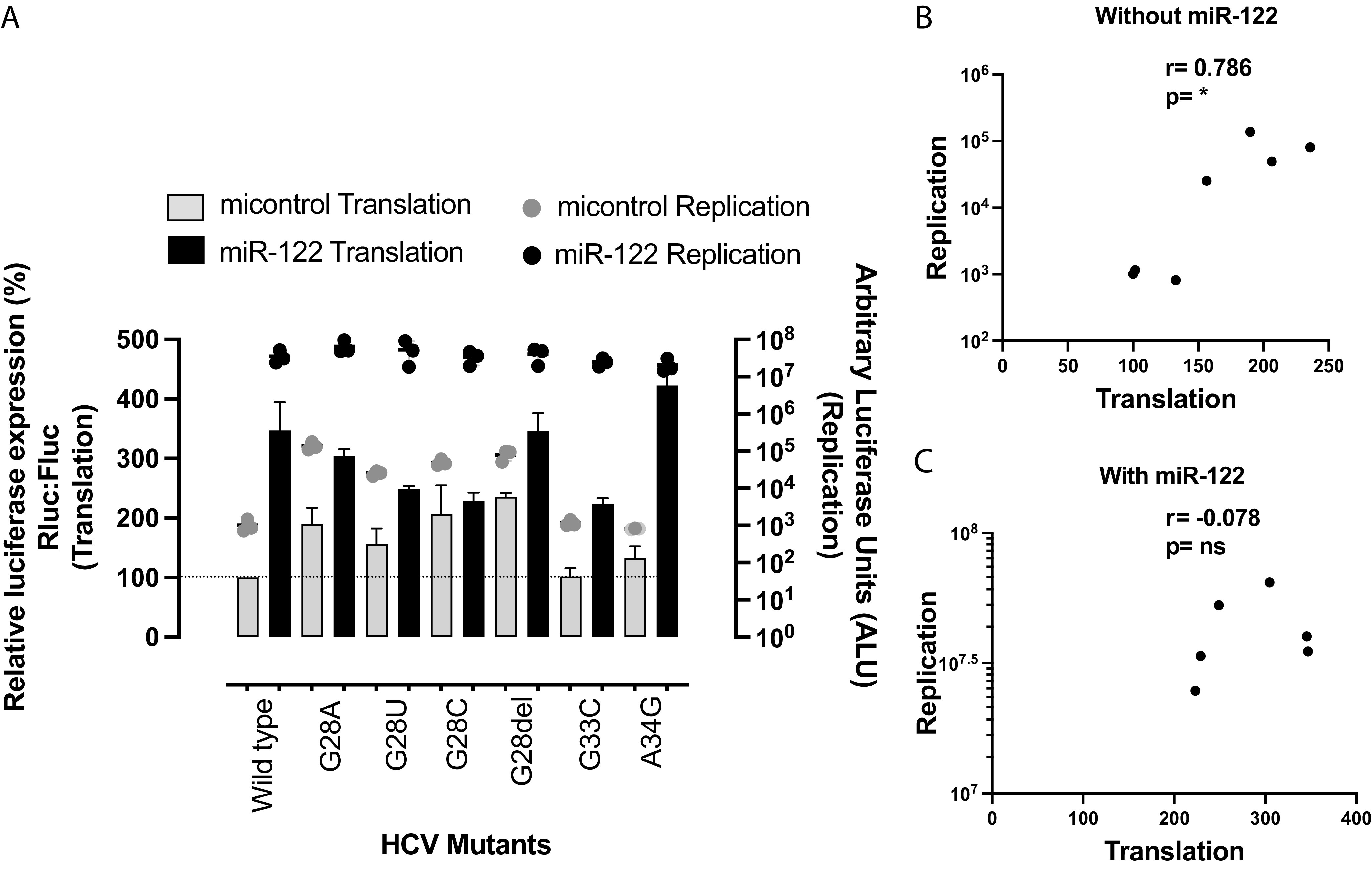
Translation and replication assays with and without miR-122: G28 mutants. Shown is a comparison of translation and replication efficiencies of G28 HCV mutants and wild-type virus in Huh 7.5 miR-122 knockout cells. The left *y* axis and bars represent HCV RNA translation, whereas the right *y* axis and the dots represent HCV RNA replication. Light gray represents viral translation/replication without miR-122, and dark gray represents viral translation/replication with miR-122. (B) Correlation of G28 HCV RNA translation and replication independently of miR-122 was analyzed using nonparametric Spearman correlation. (C) Correlation of G28 HCV RNA translation and replication in the presence of miR-122 was analyzed using nonparametric Spearman correlation. All data are presented as the averages of three or more independent experiments. Error bars indicate the standard deviations of the means, and asterisks indicate significant differences. The significance was determined by using t-distribution. ns, not significant.

To exclude the possibility that the mutations that allow for miR-122-independent replication simply enhance overall virus translation and replication ability regardless of miR-122 binding, we assessed the replication and translation of a set of mutants with and without miR-122. We expected that the mutations would primarily impact translation and replication in the absence of miR-122 and have little effect in the presence of miR-122. For these experiments, we chose viruses with mutations outside the miR-122 binding sites so that miR-122 annealing efficiency would be consistent and not a confounding factor. In Fig. 5A we show the replication and translation of the G28 mutants in the presence and absence of miR-122. We also included a wild-type control and the G33C and A34G mutants, which did not exhibit miR-122-independent replication. In the absence of miR-122, translation is correlated with replication efficiency (*r* = 0.786; *P* > 0.033), but in the presence of miR-122, translation and replication do not correlate (*r* = 0.078). Thus, the mutations enabling miR-122-independent replication do not impact the general translation and replication abilities of the viral RNAs but specifically impact translation and replication when miR-122 is absent and thus modulate the reliance on miR-122.

### Enhanced translation and genome stability can completely rescue HCV propagation in the absence of miR-122.

**FIG 6:**
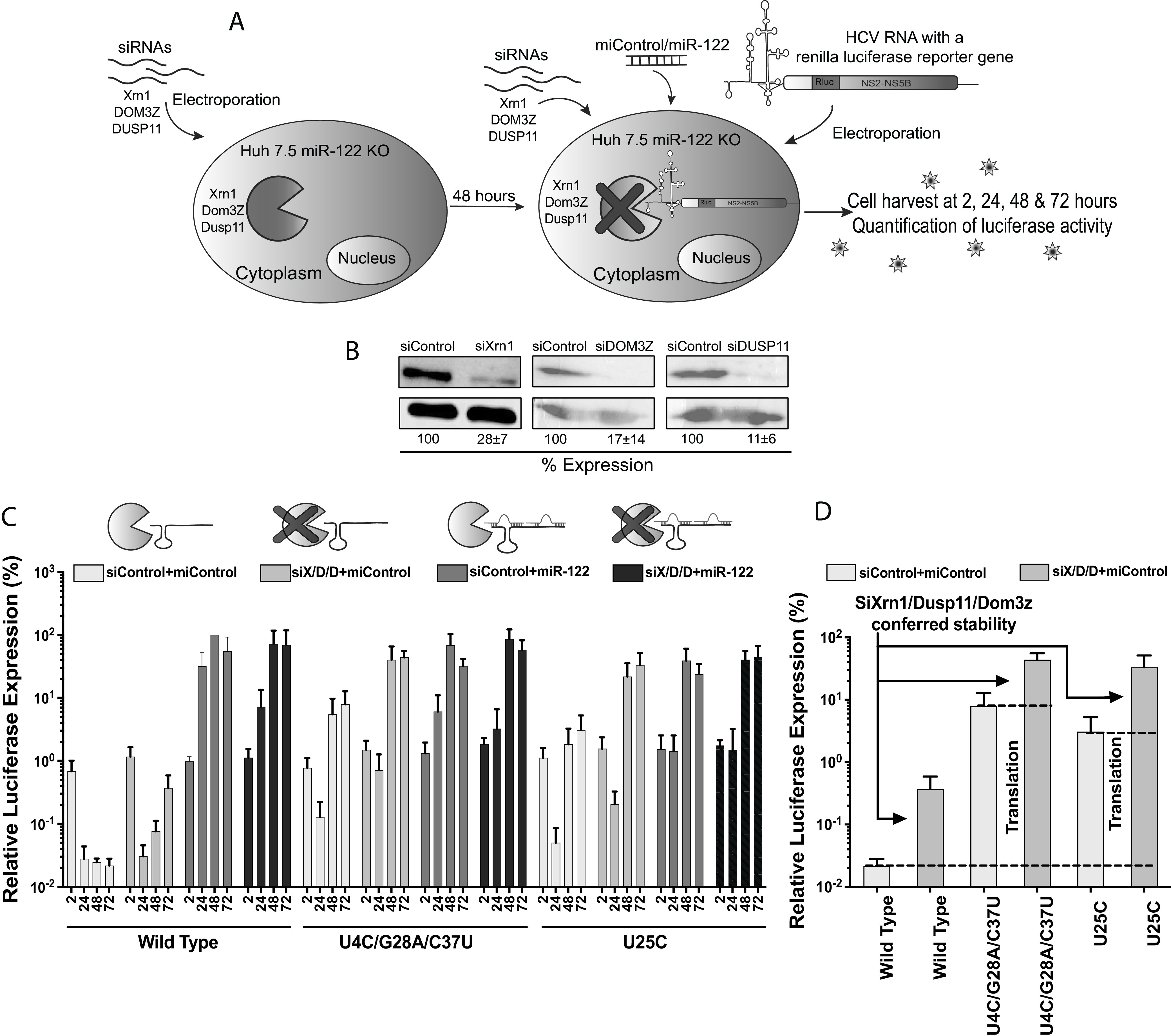
Viral genome stabilization rescues miR-122-independent replication of 5′ UTR mutants to wild-type HCV levels. (A) Graphical representation of siRNA-induced XRN1, DUSP11, and DOM3Z knockdown and HCV replication analysis in Huh 7.5 miR-122 knockout cells. Huh 7.5 miR-122 knockout cells were electroporated with XRN1-, DUSP11-, and DOM3Z-specific siRNAs, and 48 h after the first electroporation, viral RNAs along with siRNAs and miRNAs were coelectroporated into the preknockdown Huh 7.5 miR-122 knockout cells. Cells were harvested at 2 h, 24 h, 48 h, and 72 h after the second electroporation, and luciferase activity was measured as an indicator of viral propagation. (B) Western blot showing knockdown efficiency with antibodies against Xrn1, DOM3Z, and DUSP11. Percent knockdown ± standard deviation relative to siControl is shown. (C) Replication of J6/JFH-1 (p7Rluc2a) wild-type HCV, U4C/G28A/C37U HCV, and U25C HCV RNA in Huh 7.5 miR-122 knockout cells. The solid bars with different shades of gray represent different knockdown conditions. (C) The siXRN1-, siDUSP11-, and siDOM3Z-conferred stability was determined for wild-type, U4C/G28A/C37U, and U25C HCV RNA by comparing replication without knockdown (siControl) versus replication with knockdown (siS/D/D). (D) The data shown in panel C were used to determine the contribution of stability to miR-122-independent replication by comparing replication of wild-type HCV RNA in miR-122 knockout cells with (siX/D/D) and without (siControl) knockdown of XRN1, DUSP11, and DOM3Z. Similarly, the contribution of mutation-induced translation to miR-122-independent replication was determined by comparing replication of stabilized wild-type HCV RNA (WT siX/D/D) with those of stabilized mutant RNAs (U4C/G28A/C37U siX/D/D) and (U25C siX/D/D).

Roles for miR-122 in the stimulation of HCV translation and stabilization of the genomic RNA have been reported several times (18, 23, 24, 40, 41), but the relative contribution of each role to overall HCV propagation is still unknown. In this study, we have correlated translation efficiency with the ability of mutant viral RNAs to replicate independently of miR-122. However, mutation-induced miR-122-independent replication is still 10-fold lower than miR-122-dependent replication. We hypothesize that lower miR-122-independent replication efficiency is due to lack of genome stabilization by miR-122 annealing. To test this, we analyzed whether genome stabilization by other means will enhance miR-122-independent replication of the mutant genomes. These experiments also allowed for separate analyses of the relative contributions of miR-122-induced genome stabilization and genome translation to genome replication. To stabilize the genome, we knocked down cellular RNA-degrading enzymes, pyrophosphatases DOM3Z and DUSP11 and exonuclease XRN1, shown previously to enhance miR-122-independent HCV replication (25, 42). To assess the impact of genome stabilization on miR-122-independent replication of full-length viral genomes, we assessed rescued miR-122-independent replication of the wild-type versus two mutant HCV genomes (U4C/G28A/C37U and U25C) (Fig. 6A) following knockdown of XRN1, DOM3Z, and DUSP11. Protein knockdown was confirmed by Western blotting (Fig. 6B). Our results showed that knockdown of XRN1, DOM3Z, and DUSP11 rescued miR-122-independent replication of the U4C/G28A/C37U and U25C mutants to levels similar to those seen in the presence of miR-122 (Fig. 6C, mutant HCV+miControl+siDOM3Z, siXRN1, and siDUSP11 versus mutant HCV+miR-122+siControl). miR-122-independent replication of the wild-type virus was also enhanced by knockdown of XRN1, DOM3Z, and DUSP11 but did not reach miR-122-dependent levels (Fig. 6C, wild-type HCV+miControl+siXRN1, siDUSP11, and siDOM3Z versus wild-type HCV+miR-122+siControl). From this experiment, we suggest that enhanced translation derived from 5′-terminal point mutations can completely rescue viral replication to miR-122-dependent levels when the RNA is stabilized, and thus, enhanced translation and genome stabilization can completely compensate for the role of miR-122 in the viral life cycle.

We also used this assay to attempt to separate the relative impact of miR-122 on viral translation and genome stability. Knockdown of XRN1, DOM3Z, and DUSP11, which stabilizes the viral genome, increased miR-122-independent luciferase expression of wild-type, U4C/G28A/C37U, and U25C viruses almost 10-fold compared to luciferase levels without knockdown (Fig. 6D). Thus, we conclude that miR-122-induced RNA stabilization accounts for about a 10-fold increase in replication. In addition, we assessed the relative impact of translation stimulation on HCV replication by comparing luciferase levels from the wild type versus mutants in DOM3Z, XRN1, and DUSP11 knockdown cells (Fig. 6D). In this experiment, we eliminated the need for miR-122 in viral genome stabilization by knocking down DOM3Z, XRN1, and DUSP11, thus allowing the experiment to isolate the impact of the translation-enhancing mutations on virus replication. In this case, the mutant viruses replicated about 100-fold greater than wild-type virus, and we conclude that miR-122-induced translation stimulation accounts for about a 100-fold increase in HCV replication. While we acknowledge that enhanced genome stabilization will also indirectly affect virus protein expression levels, our results suggest that translation stimulation is the primary mechanism by which miR-122 promotes HCV propagation and that genome stabilization is an important but less potent mechanism. That enhanced translation and genome stabilization rescues can completely compensate for the absence of miR-122 also suggests that there may be no other roles for miR-122 in promoting HCV replication.

### HCV mutants capable of miR-122-independent replication can also replicate in Drosha knockout cells.

**FIG 7:**
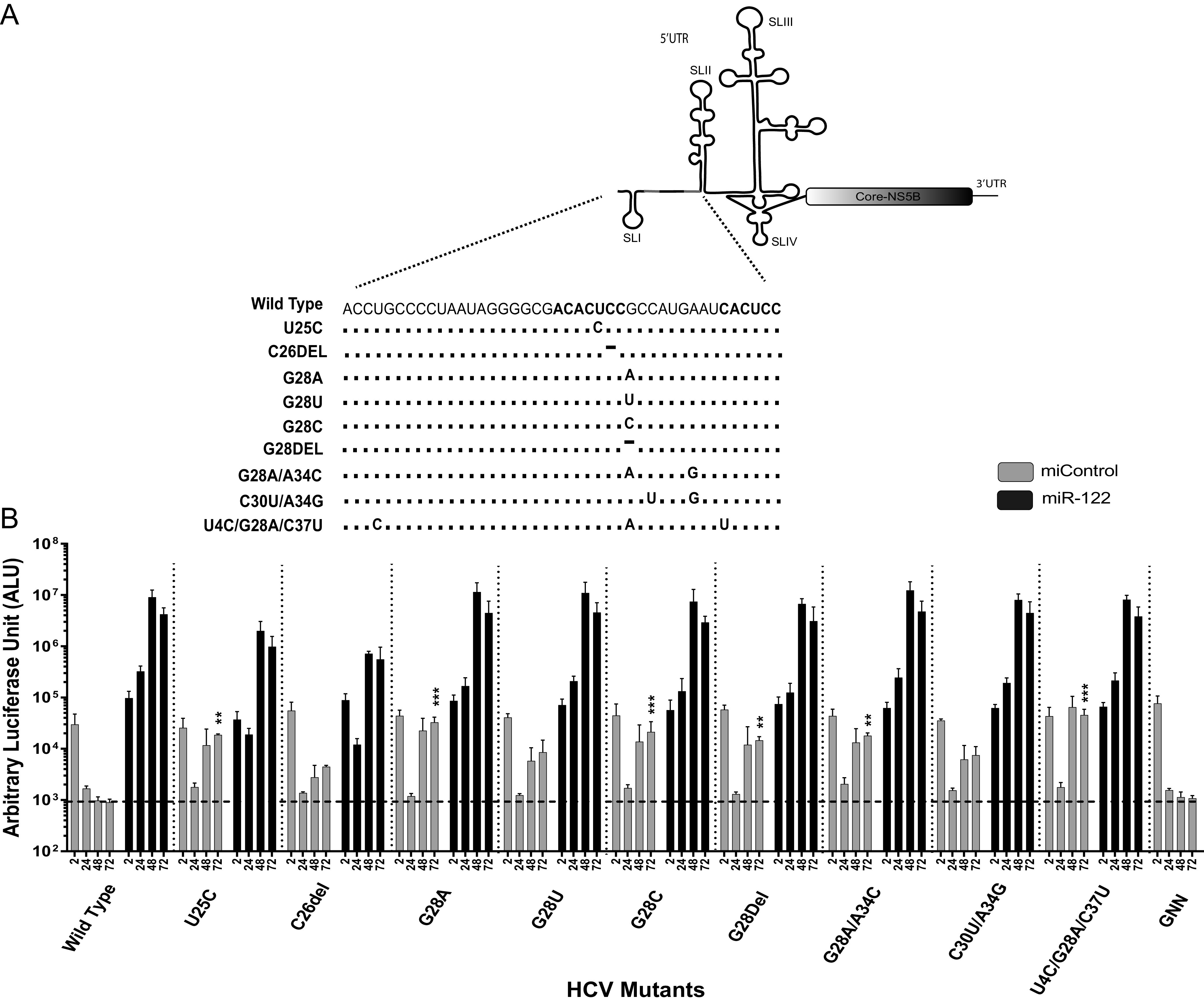
Replication of HCV mutants in Huh 7.5 Drosha knockout cells. (A) Reported mutations on the extreme 5′ UTR of the viral genome that promote virus replication independently of miR-122. The number indicates the position of the nucleotide with the mutation. (B) Transient replication of HCV mutants in Huh 7.5 Drosha knockout cells electroporated with J6/JFH-1 p7Rluc2a HCV RNA with a control miRNA (gray bars) or miR-122 (black bars). Luciferase activity of cells harvested after 72 h postelectroporation was measured, and relative luciferase activity was plotted with respect to the replication-defective mutant (GNN). All data shown are the averages of three or more independent experiments. Error bars indicate the standard deviations of the means, and asterisks indicate significant differences, as determined by one-way ANOVA (**, *P* < 0.002; ***, *P* < 0.001).

Although miR-122 is essential for HCV propagation, we and others have previously reported that full-length HCV variants with point mutations in the 5′ UTR of the genome are capable of replicating in the absence of miR-122 in miR-122 KO cells (10, 31, 33, 34). However, a report published during our studies showed that HCV replication can be supported by other miRNAs in both a miR-122-like (miR-504-3p, miR-574-5p, and miR-1236-5p) and non-miR-122-like manner (miR-25-5p and miR-4730) (36). Thus, we tested for the impact of other miRNAs on the replication of HCV mutants previously reported as capable of replicating independently of miR-122 by assessing their replication in Huh 7.5 Drosha KO cells (Fig. 7A). This cell line is devoid of Drosha and thus also devoid of cellular miRNAs generated by the canonical Drosha-dependent biogenesis pathway. Huh7.5 Drosha KO cells were electroporated with wild-type and mutant HCV RNAs, with and without the addition of miR-122. Cell extracts were harvested at 2 h, 24 h, 48 h, and 72 h postelectroporation and luciferase expression was measured as a proxy for viral RNA accumulation (Fig. 7B). Our results showed that most of the mutants previously reported to replicate independently of miR-122 also replicated in Huh 7.5 Drosha KO cells and thus independently of Drosha-dependent microRNAs. The mutants that grew least efficiently in Huh 7.5 miR-122 KO cells, the C26del, C30U/A34G, and G28U mutants, did not show significant replication in Huh 7.5 Drosha KO cells, and mutants found previously to display the most efficient miR-122-independent replication, the U25C mutant and the U4C/G28A/C37U triple mutant, replicated the most efficiently in Huh 7.5 Drosha KO cells. We confirmed that miRNAs found previously to support HCV replication, miR-504-3p, miR-574-5p, miR-1236-5p, miR-25-5p, and miR-4730, were undetectable in Huh 7.5 Drosha KO cells by reverse transcription-quantitative PCR (qRT-PCR) (data not shown). In addition, the absence of miR-504-3p, miR-1236-5p, and miR-4730 in wild-type Huh 7.5 cells was confirmed by researchers who analyzed miRNA levels in Huh 7.5.1 cells, a subclone of Huh 7.5 cells, using transcriptome sequencing (RNA-seq) (43). The ability of the mutants to replicate in Drosha KO cells had a similar trend but were less than that in miR-122 KO cells, and Drosha KO cells were also less able to support both miRNA-independent and miR-122-dependent replication (compare Fig. 2B, miR-122 KO, with Fig. 7, Drosha KO wild-type + miR-122). Thus, while we have confirmed that the HCV mutant viral RNAs are capable of replicating in an environment devoid of miRNAs generated via the Drosha biogenesis pathway, we cannot rule out a contribution of Drosha-independent small RNAs to HCV replication in miR-122 KO cells. However, the fact that miR-122-independent replication levels were similar between Drosha KO and miR-122 KO cells suggests that any impact is minor.

## DISCUSSION

In this study, we investigated miR-122-independent replication of HCV in an effort to understand the role of miR-122 in promoting the HCV life cycle. We have used several virus mutants having 5′ UTR mutations reported by us and others that support miR-122-independent replication, and our analyses suggest that translation stimulation may be the primary role for miR-122 in promoting the virus life cycle and that genome stabilization has a less potent impact but is still important. Our data also suggest that miR-122 does not have other roles in virus replication since enhancing translation and genome stabilization by other methods reinstates miR-122-dependent levels of HCV replication in the absence of miR-122 for some mutant virus genomes.

In this study, we have explored the translation efficiency of several mutants that replicate independently of miR-122 and found that all mutant viruses capable of miR-122-independent replication also had enhanced translation abilities and that the translation strength of the mutant genomes correlated with miR-122-independent replication abilities (Fig. 4). This suggests that translation stimulation by miR-122 contributes to HCV replication promotion, and mutants with enhanced translation efficiency can compensate for the lack of miR-122. This supports our previous finding that altered translation regulation may compensate for miR-122’s function in viral propagation (30) based on miR-122-independent replication of bicistronic JFH-1 subgenomic replicon (SGR) RNAs in which viral protein expression is driven by the EMCV IRES instead of the HCV IRES. Translation stimulation was proposed several years ago as a mechanism by which miR-122 promotes the HCV life cycle, but the small stimulation of translation observed, approximately 2-fold, appeared insufficient to account for the dramatic effect on virus replication. However, a recent study on translation and replication dynamics of picornavirus (positive-strand RNA virus) suggested that the efficiency of initial viral translation and production of a sufficient amount of protein to initiate genome replication are important to establish an infection, and in cases in which there is an insufficient translation and failed replication, the virus reinitiates translation for another attempt to establish an infection (44). The study also suggested that viral translation occurs at multiples phases of the viral life cycle. Hence, the seemingly small translation stimulation by miR-122 as detected in translation assays could have a significant and compounding role throughout the viral life cycle.

A second proposed mechanism by which miR-122 promotes the HCV life cycle is by stabilizing the viral genome, and our results indicate that miR-122-induced genome stabilization has a less potent impact than translation stimulation but is still important for efficient virus propagation. While we were not able to directly compare the stabilities of the mutant viral RNAs in cells, we indirectly assessed the contribution of genome stability to mutant RNA replication by analyzing it in the absence of cellular RNA degradation proteins. We found that miR-122-independent replication of mutant viruses was rescued to miR-122-dependent levels by knockdown of exonuclease XRN1 and pyrophosphatases DOM3Z and DUSP11, suggesting that genome stabilization allows mutants genomes having mutations that enhanced translation to replicate to miR-122-dependent levels. This supports the role of miR-122 in both translation stimulation and genome stabilization and the notion that wild-type levels of miR-122-independent replication can be achieved by providing these roles using alternative methods. Further, we were also able to determine the relative contributions of translation stimulation and genome stabilization to ~100-fold and ~10-fold, respectively. Although we suggest translation as a major contributor to miR-122’s function in viral propagation, and miR-122-induced genome stability is a minor but essential function in HCV propagation, we also suggest that HCV genome translation and stability are overlapping functions and one event can contribute to other. However, our data suggest a lack of an additional role of miR-122 in viral genome replication.

We and others have proposed a model that binding of miR-122 to the 5′ UTR induces the formation of the translationally active RNA IRES secondary structure (10, 11, 28, 29). The proposed model suggests that the 5′ UTR RNA structure is dynamic and forms one or more translationally unfavorable structures (SLII^Alt^) in the absence of miR-122 and that miR-122 annealing modifies the thermodynamics to favor the formation of the translationally active SLII structure. Based on extensive structure-function analyses and X-ray crystallography, nucleotides 40 to 372 of the HCV RNA form an IRES structure in solution (4, 45). Our model proposes that the 5′ terminal nucleotides 1 to 42, including the miR-122 binding sites, modulates the structure of the IRES; however, X-ray crystallography experiments were done using RNAs that lacked the 5′-terminal region containing the miR-122 binding sites (4, 45). We propose that 5′ UTR point mutants that promote miR-122-independent replication also shift the equilibrium to favor the formation of SLII, even in the absence of miR-122; in support of this hypothesis, the mutants we identified to have enhanced translation are also predicted to favor the formation of the canonical SLII prediction structures in the absence of miR-122 (10, 28) (Fig. 1 and 3). In contrast, however, some of the mutants, specifically the site 2 HCV-S2-GGCGUG, HCV-S2C-GUGAGG, and A38U mutants, were predicted to form other RNA structures, suggesting that the mechanism might be more complex. In addition, the model does not consider the roles of RNA binding proteins recruited or displaced by miR-122 which have been proposed to also modulate 5′ UTR structure and activity (28). Thus, confirmation or refinement of the model will require biophysical analyses of the RNA structures generated by these mutants and investigation of RNA protein binding.

In the process of studying miR-122-independent replication of these mutants, we have identified one 5′ UTR mutant, S2p5 (C39G), that does not replicate well even in the presence of miR-122. We speculate that this mutation may affect the structure and function of the complement strand of the 5′ UTR during genome replication. A study by Friebe and Bartenschlager showed that the RNA secondary structure of the negative strand affects viral RNA replication (12). Thus, we suggest that mutations in the 5′ UTR of the viral genome may affect miR-122 annealing and the secondary structure of both strands and this could limit the tolerance of the 5′ UTR to point mutations. Thus, we suggest that miR-122-independent replication of HCV is dictated by the multifunctional nature of the 5′ UTR and its complementary strand.

It is also interesting that while numerous HCV 5′ UTR point mutants capable of miR-122-independent replication have been isolated in the lab (Fig. 2B), few are found in patients’ samples. Only the G28A mutant was found in hepatic and extrahepatic tissue of patients as well as in cell culture, supporting miR-122-independent replication and suggesting evolutionary pressure to retain a dependence on miR-122, perhaps to restrict HCV replication to the liver. In addition, nonprimate hepaciviruses have at least one conserved miR-122 binding site on their genome, suggesting an evolutionary relationship to retain dependence on miR-122 (34, 46).

A recent report suggests that HCV replication can be supported by other miRNAs (miR-504-3p, miR-574-5p, and miR-1236-5p) in a miR-122-like manner and by miR-25-5p and miR-4730 in a non-miR-122-like manner (36). To investigate the possible effects of these miRNAs on our miR-122-independent HCV replication models, we assessed replication of several 5′ UTR mutants in Huh 7.5 Drosha KO cells, cells lacking the canonical miRNA biogenesis pathway and thus lacking the miRNAs listed above and miR-122. We found that the mutant viruses replicating in the absence of miR-122 could also replicate in Huh 7.5 Drosha KO cells, suggesting that replication is not supported by these miRNAs in our assays. However, we cannot exclude the possibility that miR-122-independent replication might be supported by small RNAs generated by noncanonical pathways such as mirtrons, snoRNA-derived miRNAs, and tRNA-derived miRNAs (47, 48).

In conclusion, we have established that viral variants that replicate independently of miR-122 display enhanced translation and that genome replication in the absence of miR-122 can be completely rescued when both translation and genome stability are addressed by other means. Finally, we show evidence that translation stimulation is a major function of miR-122-induced promotion of HCV, whereas genome stability has a secondary function.

## MATERIALS AND METHODS

### Cells.

Human hepatoma Huh 7.5 (49) and Huh 7.5 Drosha KO cells were kind gifts from C. M. Rice (50). The Huh-7.5 miR-122 KO cell line was gifted by Matthew Evans (31). All the cells were cultured in Dulbecco’s modified Eagle medium (DMEM) supplemented with 10% fetal bovine serum, 0.1 nM nonessential amino acids (Wisent, Montreal, Canada), and 100 μg/mL of penicillin-streptomycin (pen-strep; Invitrogen, Burlington, ON, Canada).

### Plasmids.

Plasmid pJ6/JFH-1 Rluc (p7-Rluc2a), including full-length viral sequences derived from the J6 (structural proteins) and JFH-1 (nonstructural proteins) isolates of HCV and a *Renilla* luciferase reporter (Rluc) gene directly downstream of the p7 gene was provided by C. M. Rice (herein called pJ6/JFH-1 RLuc) (51). The replication-defective mutant has a GAA-GNN mutation in the viral polymerase active site, pJ6/JFH-1 Rluc GNN. All site 1, site 2, and other 5′ UTR mutants were created by site-directed mutagenesis PCR of the PBSKS+ vector with a cloned fragment of the pJ6/JFH-1 Rluc plasmid from EcoRI to KpnI. After confirming the mutation(s) by sequencing, the EcoRI-to-KpnI fragment from the PBSKS+ plasmid was swapped into pJ6/JFH-1 Rluc WT plasmid to generate mutant HCV variants with mutations in the 5′ UTR. Replication-defective mutants of all the viral variants were created by inserting the 5′ UTR mutant EcoRI-to-KpnI fragment into pJ6/JFH-1 Rluc GNN. A pT7 plasmid encoding an mRNA with a firefly reporter gene was used to generate control mRNA for translation assays (Promega, Madison, WI, USA), herein referred to as pT7 Fluc. Plasmid pSGR JFH-1 Fluc contains bicistronic JFH-1-derived subgenomic replicon (SGR) cDNAs with a firefly luciferase reporter (52). pSGR JFH-1 Fluc U4C/G28A/C37U plasmid was cloned by swapping the EcoRI-AgeI fragment from the pJ6/JFH-1 Rluc (p7-Rluc2a) plasmid containing the U4C/G28A/C37U mutation to pSGR JFH-1 Fluc. Plasmid pSGR JFH-1 Fluc was a gift from Takaji Wakita.

### microRNA and siRNA sequences.

The sequence of synthetic miR-122 guide strand was UGGAGUGUGACAAUGGUGUUUGU, and that of the passenger strand was AAACGCCAUUAUCACACUAAAUA. miControl is a mutated version of miR-122 in which sites 2 to 8 on the guide strand were converted to their complements, guide strand UAAUCACAGACAAUGGUGUUUGU and the passenger strand AAACGCCAU UAUCUGUGAGGAUA (24). siXRN1 (si29015; 5′-GAG AGU AUA UUG ACU AUG ATT-3′), siControl (5′-GAA GGU CAC UCA GCU AAU CAC Ttc-3′) (24), and siDOM3Z SMARTpool siGENOME were obtained from Dharmacon (Lafayette, CO, USA); siDUSP11 (AM16708) was purchased from Thermo Fisher Scientific (Waltham, MA, USA).

### *In vitro* transcription.

pJ6/JFH-1 Rluc plasmids were linearized by digestion with XbaI and mung bean nuclease, and *in vitro* transcription was performed using the MEGA Script T7 high-yield transcription kit (Life Technologies, Burlington, ON, Canada). To generate T7 Fluc mRNA, pT7 Fluc plasmid was linearized with XmnI and an mMessage mMachine mRNA synthesis kit (Life Technologies) was used for *in vitro* transcription all of the transcription process was performed using the manufacturer’s suggested protocol.

### Electroporation of cells.

Electroporations were carried out as described by Thibault et al. (24). Both Huh7.5 miR-122 KO cells and Huh 7.5 Drosha KO cells were electroporated using the following conditions: 225 V, 950 μF, 4 mM, and ∞ Ω.

### Transient HCV replication assay.

On day 0, Huh-7.5 cells were coelectroporated as described previously (41) with 5 μg of J6/JFH-1 Rluc wild-type RNA or mutant viral RNAs and 60 pmol of miR-122 or control microRNA into approximately 6.0 × 10^6^ cells in 400 μL of Dulbecco’s phosphate-buffered saline (PBS). Electroporated cells were resuspended in 4 mL of cell culture medium, and 500 μL of cells was plated in 6-well dishes and incubated at 37°C to be harvested at different time points. The electroporation protocol for Huh 7.5 Drosha KO cells was similar to described above; however, a total of 750 μL of resuspended electroporated cells was plated onto the 6-well dishes instead of 500 μL.

### Transient HCV replication assay with preknockdown.

Exonuclease (XRN1) and pyrophosphatase (DOM3Z and DUSP11) preknockdown assays were performed as described previously (25). At 48 h before day 0, approximately 6.0 × 10^6^ cells in 400 μL of Dulbecco’s PBS were electroporated with 60 pmol of each small interfering RNA (siRNA) together for preknockdown and were plated in 15-cm tissue culture dishes, two cuvettes per dish. siRNA-treated cells were incubated for 2 days at 37°C. On day 0, cells were collected from the dishes, prepared as described for electroporation (two cuvettes’ worth from each dish) and then electroporated with viral RNA, siRNAs, and microRNAs and plated as described above.

### Western blotting.

Western blotting was performed as described previously. The blots were probed with primary antibodies: rabbit polyclonal anti-DOM3Z (Cedarlane), rabbit polyclonal anti-DUSP11 (ProteinTech), rabbit polyclonal anti-Xrn1 (Cell Signaling Technology), and mouse anti-actin (Abcam). Subsequently, the blots were probed with secondary goat anti-mouse or goat anti-rabbit conjugated with horseradish peroxidase (Jackson ImmunoResearch Laboratories, West Grove, PA, USA) and visualized using enhanced chemiluminescence (GE Healthcare, Mississauga, ON, Canada).

### Luciferase assay.

To measure luciferase expression, cells were washed with Dulbecco’s phosphate-buffered saline and the lysate was harvested by using 100 μL of passive lysis buffer. *Renilla* or dual-luciferase kits (Promega, Madison, WI, USA) were used to measure luciferase as suggested by the manufacturer’s protocols. A total of 10 μL of lysate was mixed with the appropriate luciferase assay substrate and light emission was measured by using a Glomax 20/20 luminometer (Promega).

### Translation assay.

A total of 10 μg of J6/JFH-1 Rluc2a GNN viral RNA (wild type and mutant) and 1 μg of control T7 Fluc mRNA were coelectroporated in Huh 7.5 miR-122 KO cells. Cells were harvested at 2 h postelectroporation and a dual-luciferase test was done to analyze Rluc and Fluc expression.

### MicroRNA analysis.

MicroRNA expression was measured using qRT-PCR. miRCURY LNA miRNA PCR assays (Qiagen, Toronto, ON, Canada) (miR-25-5p, miR-320a-3p, miR-504-3p, miR-574-5p, miR-877-5p, and miR-1236-5p) and TaqMan MicroRNA assays (Applied Biosystems, Waltham, MA, USA) (miR-122 and miR-4730) were used as per the manufacturers’ instructions. Total RNA was extracted and purified from cells using TRIzol.

### Secondary-structure prediction.

5′ UTR nucleotides 1 to 117 of the positive strand and 3′ UTR nucleotides 1 to 117 of the negative strand of the HCV genome were used for RNA structure prediction. RNA prediction software “RNA structure” from Matthews lab at https://rna.urmc.rochester.edu/index.html was used for RNA structure prediction (39). Dot-bracket files were generated using fold and bifold algorithms for predicting the structure of a single RNA and two interacting RNAs, respectively. VARNA (VARNA GUI applet) was used to generate the folded RNA images from the dot-bracket files (53).

### Data analysis.

All experiment results are displayed as averages from at least three independent experiments. Error bars indicate standard errors of the mean (SEM). Statistical analyses were performed using Prism 7.4 (GraphPad, San Diego, CA, USA); statistical significance was determined by the tests indicated in each figure legend.
